# Differential Function of Melatonin MT_1_ and MT_2_ Receptors in REM and NREM Sleep

**DOI:** 10.3389/fendo.2019.00087

**Published:** 2019-03-01

**Authors:** Gabriella Gobbi, Stefano Comai

**Affiliations:** ^1^Neurobiological Psychiatry Unit, Department of Psychiatry, McGill University, Montreal, QC, Canada; ^2^San Raffaele Scientific Institute and Vita Salute University, Milan, Italy

**Keywords:** melatonin, MT_1_ receptor, MT_2_ receptor, sleep, REM, NREM

## Abstract

The pathophysiological function of the G-protein coupled melatonin MT_1_ and MT_2_ receptors has not yet been well-clarified. Recent advancements using selective MT_1_/ MT_2_ receptor ligands and MT_1_/MT_2_ receptor knockout mice have suggested that the activation of the MT_1_ receptors are mainly implicated in the regulation of rapid eye movement (REM) sleep, whereas the MT_2_ receptors selectively increase non-REM (NREM) sleep. Studies in mutant mice show that MT_1_ knockout mice have an increase in NREM sleep and a decrease in REM sleep, while MT2 knockout mice a decrease in NREM sleep. The localization of MT_1_ receptors is also distinct from MT2 receptors; for example, MT_2_ receptors are located in the reticular thalamus (NREM area), while the MT_1_ receptors in the Locus Coeruleus and lateral hypothalamus (REM areas). Altogether, these findings suggest that these two receptors not only have a very specialized function in sleep, but that they may also modulate opposing effects. These data also suggest that mixed MT_1_-MT_2_ receptors ligands are not clinically recommended given their opposite roles in physiological functions, confirmed by the modest effects of melatonin or MT_1_/MT_2_ non-selective agonists when used in both preclinical and clinical studies as hypnotic drugs. In sum, MT_1_ and MT_2_ receptors have specific roles in the modulation of sleep, and consequently, selective ligands with agonist, antagonist, or partial agonist properties could have therapeutic potential for sleep; while the MT_2_ agonists or partial agonists might be indicated for NREM-related sleep and/or anxiety disorders, the MT_1_ agonists or partial agonists might be so for REM-related sleep disorders. Furthermore, MT_1_ but not MT_2_ receptors seem involved in the regulation of the circadian rhythm. Future research will help further develop MT_1_ and/or MT_2_ receptors as targets for neuropsychopharmacology drug development.

## Sleep, Sleep Architecture, and Sleep Disorders

Following Tononi and Cirelli's synaptic homeostasis hypothesis (1), sleep is the price the brain pays for plasticity. Indeed, during waking, the learning process requires the strengthening of connections throughout the brain. This process increases cellular need for energy and supplies, decreases signal-to-noise ratios, and saturates learning. During sleep, cerebral spontaneous activity renormalizes the net synaptic strength and restores cellular homeostasis. This activity of synapses during sleep may also explain the benefits of sleep on memory acquisition, consolidation, and integration (1).

In mammals, physiological sleep is comprised of two distinct states called rapid-eye movement (REM) sleep and non-REM (NREM) sleep that alternate through the night in a cyclical fashion. REM occurs in short periods, characterized by a decrease in muscle tone and associated with a profound sympathetic activation, including increased heart rate, breathing, blood pressure, and temperature. NREM periods are longer and are associated with a parasympathetic activation, consisting of low blood pressure, low heart rate, and decreased temperature. While structured dreams occur mostly in REM, non-structured and bizarre dreams occur in NREM. In adults, about 75–80 percent of total time spent in sleep is spent in NREM sleep while the remaining 20–25 percent occurs in REM sleep. During the night, adult subjects usually experience four to five NREM to REM sleep cycles. Interestingly, newborns spend more time in REM, and the time spent in NREM increases progressively over the years at the expense of REM.

NREM sleep is divided into progressively deeper stages—named stage N1, stage N2, and stage N3—that can be distinguished based on specific electroencephalogram (EEG) traits [for details on this topic, which is beyond the aim of this review, please see Atkin et al. (2) and Iber et al. (3)]. However, it is important to highlight that stage N3, commonly referred to as slow wave sleep (SWS) during which there is deep or delta-wave sleep, seems important for cerebral restoration and recovery, the maintenance and consolidation of memory (4), and metabolic regulation (5). As a consequence, disturbances in the duration and architecture of sleep is often associated with next-day impairments in conducting daily activities and, if not treated, can be closely linked to many neurological and psychiatric disorders (6–8). The lack or the disruption of sleep, known as “insomnia,” is a common public health problem, with a prevalence ranging from 11 to 16% (9).

The publication of the 5th edition of the *Diagnostic and statistical manual of mental disorders* (DSM-V) (10) fundamentally changed the landscape of sleep medicine and the diagnosis of insomnia. The DSM-IV distinguished primary insomnia [characterized by a difficulty to initiate or maintain sleep for at least 1 month, with associated daytime fatigue, significant distress or social impairment (9, 10)] from insomnia secondary to another diagnosis (including major depressive disorder and generalized anxiety disorder). Instead, the DSM-V has eliminated primary insomnia as a diagnosis in favor of “insomnia disorder,” which may occur alongside other diagnoses like major depressive disorder. This revised definition obliges the clinician to treat insomnia as a distinct mental condition, even if it may be present with other mental disorders (2).

Insomnia is frequent in people suffering from major depression, with alterations in sleep neurophysiology, notably decreased SWS, reduced REM latency and increased REM density. Increased REM density has also been observed in eating disorders, narcolepsy, presenile dementia, and other neuropsychiatric diseases (11).

Besides “insomnia disorder,” mostly characterized by a decrease in NREM quantity and longer latency to sleep (first episode of NREM), the DSM-V, like DSM-IV, proposes a specific classification for REM sleep behavior disorders. REM sleep behavior disorders are characterized by recurrent episodes of arousal during sleep associated with vocalization and/or complex motor behaviors that arise during rapid eye movement (REM) sleep, confusion or disorientation on waking from these episodes, co-presence of REM sleep without atonia on polysomnographic recordings, and/or history of synucleinopathy diagnosis (e.g., Parkinson's disease, multiple system atrophy).

From a pharmacological point of view, it is thus important that a drug used to treat insomnia or sleep-related disorders not only acts on the duration of sleep but also preserves the physiological sleep architecture. Unfortunately, most of the currently available hypnotics considerably alter the physiological sleep architecture (2). In addition, official medicine has not yet recognized guidelines for specific treatment of “NREM disorders” vs. “REM disorders,” and hypnotics are non-differentially used for both conditions.

## Melatonin and Sleep: Preclinical and Clinical Findings

Currently used hypnotic drugs, such as benzodiazepines and thier derivates (i.e., zopiclone), act mostly on the GABAergic system, increasing SWS and decreasing REM sleep, thus altering the sleep architecture (2). This can result in next-day cognitive impairments and may also lead to abuse. Antidepressants, such as tricyclics and selective serotonin reuptake inhibitors (SSRIs) mostly reduce REM density, with little or no effect on SWS. The catecholamine releaser bupropion increases REM and has no effect on SWS (2, 12, 13). To develop new effective hypnotic drugs that selectively increase SWS without altering REM density and the whole sleep architecture therefore remains a scientific and medical challenge.

The physiological effects of melatonin (N-acetyl-5-methoxytryptamine, MLT) in the brain result from the activation of high-affinity (Ki ≈ 0.1 nM), G protein-coupled receptors, referred to as MT_1_ and MT_2_. Activation of both receptors mainly activates G_i_ proteins with inhibition of adenylyl cyclase and subsequent decrease of intracellular cAMP levels. Detailed information on the molecular signaling pathways activated by melatonin receptors is beyond the scope of the aim of the present work and can be found in the reviews by Dubocovich et al. (14), Jockers et al. (15), and Oishi et al. (16). However, of interest, recent lines of research have indicated that melatonin receptors can form abundant MT_1_/MT_2_ hetero-oligomers and that they can both heteromerize with other receptors, including the serotonin 5-HT_2C_ (17). Importantly, from both neurobiological and pharmacological perspectives, these heteromers display functional properties different from those of the corresponding homomers (17). For example, in the MT_2_/5-HT_2C_ heteromer, melatonin binding induces the activation of G_q_ signaling through a transactivation of the serotonergic receptor caused by conformational changes of the MT_2_, which is normally not coupled to a G_q_ (17).

Due to the lack of selective ligands for MT_1_ and MT_2_ receptors, the respective roles of these receptors in brain function and in particular in sleep regulation remain unclear.

The neuromodulator MLT is synthesized by the pineal gland and has been reported to have hypnotic effects on humans, although these results are still controversial (18–21). Meta-analysis on the effects of melatonin indeed suggest that melatonin has a soporific effect, helping people to fall asleep, but has no effects on sleep maintenance and sleep quality (18, 19).

Similarly, in laboratory animals, several studies have demonstrated that MLT reduces time to sleep onset and increases SWS and REM (22–24), effects that would be blocked by the GABA_A_ receptor antagonists flumazenil and picrotoxin (24). Others have suggested that MLT regulates REM, since lesioning of the pineal gland or the inhibition of MLT synthesis reduce REM density during light and dark periods (25–27). The effects of MLT (3–5 mg/kg) in Djungarian hamsters and rats (both nocturnal animals) were short lasting and depended on the time of day. MTL prolonged sleep latency in the late light period, enhanced sleep fragmentation in the early light period, and elevated body temperature. REM sleep was reduced when hamsters were treated with MLT after the late light period and when rats were treated after dark onset. These indicate that MLT induces changes that are typical for the dark period of each species, i.e., wakefulness in the nocturnal Djungarian hamster and rat, and sleepiness in diurnal animals (28).

Electrophysiological recordings in monkeys have indicated that MLT has only a weak and transient effect on sleep in these species (29, 30), decreasing the latency of the first episode of sleep (31).

Five non-selective MT_1_/MT_2_ agonists—ramelteon (S)-*N*-[2-(1,6,7,8-tetrahydro-2*H*-indeno[5,4-*b*]furan-8-yl)ethyl]propionamide, tasimelteon (VEC-162; structure not disclosed), TIK-301 (β-methyl-6-chloroMLT; *N*-[(2R)-2-(6-chloro-5-methoxy-1*H*-indol-3-yl)propyl]acetamide), agomelatine (N-[2-(7-methoxynaphthalen-1-yl)ethyl]acetamide) and piromelatine (N-[2-(5-methoxy-1H-indol-3-yl)ethyl]-4-oxopyran-2-carboxamide—have been tested in different species for potential use in insomnia. Ramelteon seems to have insignificant effects on sleep in rats (32), monkeys (31), and cats (29). Agomelatine, on top of being a non-selective MT_1_/MT_2_ agonist, also acts as an antagonist at the level of 5-HT_2C_ receptors. Agomelatine increases NREM and REM sleep in rats but only if administered shortly before the dark phase (active phase for rodents), but not during the light phase (inactive phase for rodents) (32). In the same experiment, melatonin increased REM sleep, which was followed by an increase in wakefulness (32). Tobler et al. (30) found that melatonin and agomelatine (S-20098) reduced the power density in NREM sleep in the low frequency range (1–8 Hz), but did not affect the vigilance states and brain temperature. Sleep data with tasimelteon and TIK-301 in rats are lacking (33).

A summary with the preclinical data investigating the effects of melatonin and non-selective MT_1_/MT_2_ agonists on the sleep/wake cycle of rats is reported in Table 1.

**Table 1:** Acute effects of melatonin, non-selective MT_1_/MT_2_ receptors agonists, and selective MT2 receptors partial agonists, agonists and antagonists on sleep/wake stages of rats during the 24-h light/dark cycle.

	**Latency to NREM sleep**	**NREM sleep duration**	**REM sleep duration**	**Wakefulness duration**
Melatonin	ø (32) ↓ (23) n.r. (30)	ø (23, 30) Dark phase: ↓↑ depending on time after administration (32) Light phase: ø (32)	ø (23, 30) Dark phase:↓↑ depending on time after administration (32) Light phase: ø (32)	ø (23, 30) Dark phase: ↑ depending on time after administration (32) Light phase: ø (32)
Non-selective MT_1_/MT_2_ receptors agonist UCM793 (34)	ø	ø	ø	ø
Non-selective MT_1_/MT_2_ receptors agonist Agomelatine	n.r. (30) ø (32)	ø (30) ↑ Dark Phase (32) ø light phase (32)	ø (30) ↑ Dark Phase (32) ø light phase (32)	ø (30) ↓ Dark Phase (32) ø light phase (32)
Non-selective MT_1_/MT_2_ receptors agonist Ramelteon	↓ (35) ø (32)	↑ (35) Dark phase: transient ↑ 4 h after administration (32) Light phase: ø (32)	ø (35) Dark phase: transient ↑ 4 h after administration (32) Light phase: ø (32)	↓ (35) Dark phase: transient ↑ 4 h after administration (32) Light phase: ø (32)
Selective MT_2_ receptors partial agonists UCM765 (34) and UCM924 (23)	↓	↑	ø	↓
Selective MT_2_ receptors agonist IIK7 (36)	↓	↑	ø	n.r.
Selective MT_2_ receptors antagonist4P-PDOT (34)	ø	ø	ø	ø

*↓, decrease; ↑, increase; ø, no change; n.r., not reported*.

Ramelteon (37–39), tasimelteon (40), and TIK (41, 42) have also been tested in humans for the treatment of insomnia. All three significantly reduced the latency to sleep in humans, but their effect on total sleep time was minimal.

In particular, the non-selective MT_1_-MT_2_ receptor ramelteon decreases the latency of sleep but not the whole duration (39, 43) and for this reason was approved by the Food and Drug Administration (FDA, United States) but not the European Medicines Evaluation Agency (EMEA) because “.*. the difference in the time taken to fall asleep between patients taking Ramelteon and those taking placebo was considered to be too small.…When other aspects of sleep were considered, Ramelteon did not have any effect*.” (https://www.ema.europa.eu/medicines/human/withdrawn-applications/ramelteon, consulted on November 1, 2018).

Similarly, the non-selective agonist tasimelteon (VEC-162) was effective for treatment of transient insomnia associated with shifted sleep and wake time (44) and was developed as an orphan drug for the treatment of Non-24-H Sleep-Wake Disorder, but not for insomnia. The EMEA approved tasimelteon for the same condition but only in completely blind people (https://www.ema.europa.eu/documents/assessment-report/hetlioz-epar-public-assessment-report_en.pdf, consulted on November 1, 2018).

Agomelatine was also approved by the EMEA as an antidepressant, but a recent meta-analysis has pointed out its low effects compared to other classes of antidepressants (45), some clinical evidence has shown that agomelatine could be efficacious in sleep disorder (46), especially if associated with depression (47).

Piromelatine is a MT_1_and MT_2_ agonist with agonism also at 5-HT_1A/1D_ receptors (48). Piromelatine was shown to have both hypnotic and antinociceptive effects by electroencephalogram (EEG) recordings in an animal model of neuropathic pain, partial sciatic nerve ligation (PSL) (49). It increases NREM sleep and decreases wakefulness in PSL mice, but the effect could be blocked by preadministration of a melatonin receptor antagonist, a 5-HT_1A_ receptor antagonist, or an opiate receptor antagonist (49), demonstrating a lack of selectivity for the melatonin receptors.

In 2013, Neurim Pharmaceuticals Ltd announced positive results from a phase II randomized clinical trial (*N* = 120) of piromelatine for the treatment of primary insomnia (50). Active treatment with piromelatine at 20 or 50 mg/d over 4 weeks resulted in significantly improved wake after sleep onset (WASO). However, the primary outcome of latency to persistent sleep was not significant when compared with the placebo (https://clinicaltrials.gov/ct2/show/results/NCT01489969) and consequentiality, the company did not further develop piromelatine for insomnia. The Clinicaltrials.gov database lists a study currently recruiting patients entitled “Safety and Efficacy of Piromelatine in Mild Alzheimer's Disease Patients (ReCOGNITION),” https://clinicaltrials.gov/ct2/show/NCT02615002, indicating that the compound will be primarily developed for cognition and not for sleep (a secondary outcome of the study).

Altogether, these animal and clinical studies have pointed out the equivocal effects of non-selective agonists on sleep duration, despite the undoubted evidence that MLT receptors are implicated in sleep regulation and circadian rhythms.

## MT_1_ and MT_2_ Receptors and Sleep

Sleep is regulated by two processes, the sleep/wake homeostasis and the circadian clock (51). In mammals, the master circadian clock is located in the suprachiasmatic nucleus (SCN) of the hypothalamus. The SCN receives direct inputs about the external environmental day/night cycle from the retina via the retino-hypothalamic tract, and then accordingly, controls the synthesis of melatonin by the pineal gland. In turn, melatonin controls the SCN activity via a feedback mechanism involving MT_1_ and MT_2_ receptors located in the SCN (52).

### Insights From Pharmacological Studies With MT_2_ Selective Ligands

In our lab, we used electroencephalogram (EEG) and electromyogram (EMG) recordings in rats for 24 h to examine the effects of the selective MT_2_ partial agonists UCM765 and UCM924 on sleep in comparison with diazepam, melatonin and the non-selective MT_1_-MT_2_ agonist UCM793 (23, 34).

We observed that UCM765 decreased the latency to the first episode of NREM sleep and increased the total amount of NREM sleep, in particular during the light (non-active) phase. We then compared the effects of UCM765 with those of the clinically-used hypnotic drug diazepam, and observed similar effects on both latency to the first episode and duration of NREM sleep. However, we found that, unlike diazepam, UCM765 did not induce a significant suppression of delta power activity during NREM sleep (34). Similar to melatonin, the MT_1_/MT_2_ non-selective agonist UCM793 did not produce significant effects on sleep stages (34), suggesting that the MT_2_ receptor subtype is probably the one mainly involved in the regulation of NREM sleep but the MT1 may counterbalance the MT_2_-mediated effects. This hypothesis is also supported by the fact that knockout mice for both MT_1_ and MT_2_ receptors (53), as well as pinealectomized rats (54), do not show impairments of NREM and REM sleep duration. However, we cannot exclude the possibility that melatonin acts on sleep through mechanisms independent of MT_1_/MT_2_ activation. Indeed, unlike the non-selective MT_1_/MT_2_ agonist UCM793 (34), melatonin significantly reduced the latency to NREM sleep onset but not to REM sleep onset (23). Evidence has shown that melatonin can interact with other neurotransmitter systems implicated in the neurobiology of sleep (2) including alpha-7 nicotinic (55, 56) or GABA (24) receptors and the release of serotonin (57, 58).

Targeting the MT_2_ receptors with the MT_2_ agonist IIK7 also selectively increased the duration of NREM sleep without affecting REM sleep, although the overall effects appear to be only transient (36). Future studies should investigate possible differential effects on NREM sleep produced by partial and full agonists toward the MT_2_ receptors. There is not yet a clear understanding of whether and how MT_1_ and MT_2_ receptors may desensitize upon stimulation by exogenous melatonin/selective ligands and according to the daily fluctuating levels of endogenous melatonin. Nonetheless, it appears that the hypnotic effects of MT_2_ partial agonists may be superior to those of MT_2_ full agonists, because the former would avoid the rapid desensitization induced by the full agonist. The higher pharmacological efficacy of MT_2_ partial agonists over melatonin has also been found when comparing their analgesic effects in preclinical models of neuropathic pain (59, 60).

Given the paucity of selective MT_1_ receptor ligands, only one pharmacological study has explored the effects of MT_1_ receptor activation/inhibition upon the sleep stages, indicating a possible selective effect of MT_1_ receptor selective ligands on REM sleep activation (61).

### Validating MT_2_ Receptors as a Target to Selectively Promote NREM Sleep

UCM765 and UCM924 have shown high selectivity and affinity toward MT_2_ receptors and low affinity toward a panel of many other receptors (59) known to be involved in sleep (2, 62). Furthermore, the role of MT_2_ receptors in the observed effects of these two drugs on sleep has been validated using both pharmacological and genetic approaches. The MT_2_ receptor antagonist cis-4-phenyl-2-propionamidotetralin (4P-PDOT) is a reference compound exhibiting good binding affinity for the human cloned MT_2_ receptor (p*K*_i_ = 8.8), and a selectivity for the MT_2_ receptor at least 100 fold that of the MT1 subtype (63). In order to test the hypothesis that the promotion of NREM sleep is MT_2_-mediated, we administered 4P-PDOT (10 mg/kg, a dose not affecting sleep stages) 10 min prior to UCM765, and found that 4P-PDOT completely blocked the effects of UCM765 on NREM sleep duration (34). UCM765 was also tested in MT_2_KO, and, unlike in wild-type control mice, the compound did not enhance NREM sleep in the MT_2_KO animals. These data strongly confirm the important role of MT_2_ receptors in modulating NREM sleep.

### Insights From MT_1_, MT_2_ and Double MT_1_-MT_2_ Receptors Knockout Mice

The role of melatonin receptors in sleep has also been investigated by taking advantage of knockout mice for MT_1_ and/or MT_2_ receptors.

Quite surprisingly, the lack of both MT_1_ and MT_2_ receptors did not significantly affect the amount of NREM and REM sleep during the 24 h (53). In contrast, a slight but significant increase in the time of wakefulness during the 24 h was present (53). These findings suggest that the lack of both melatonin receptors only minimally influences the two sleep stages, in agreement with the finding that also the lack of melatonin (their physiological ligand) due to a pinealectomy does not significantly affect the duration of sleep (54).

In keeping with the pharmacological studies reported above, the genetic inactivation of only one of the two melatonin receptor subtypes instead produces significant effects on the sleep stages. MT_2_KO mice display a significant reduction of NREM sleep duration during 24 h, with the decrease mainly due to an effect occurring during the light (inactive) phase (34, 53). No effects on REM sleep duration have been observed in MT_2_KO mice (34, 53). These findings in MT_2_KO mice corroborate pharmacological findings with MT_2_ agonists/partial agonists (23, 34, 36) demonstrating a selective role of MT_2_ receptors in regulating NREM sleep.

In MT_1_KO mice a significant decrease in the duration of REM sleep has been observed (34, 53), suggesting a central role for MT_1_ receptors in REM sleep regulation. In contrast, the possible involvement of MT_1_ receptors in NREM sleep remains unclear. While in rats there is concordance among studies on how to score sleep stages, different protocols have been used in mice. In particular, sleep is scored using either 4 or 10 s epochs (64). However, given that in mice very short episodes of REM sleep are present, the 4 s epoch seems probably the best way to score sleep in mice (64). In keeping with this rationale, we found that the duration of NREM and REM sleep in MT_1_ mice can slightly differ depending on the 4 or 10 s methodological approach. Using 4 s epochs, we found no change in NREMS in MT_1_KO mice compared with WT control animals. In contrast, using 10 s epochs we found a slight but significant increase of NREM sleep during the dark/active phase in MT_1_KO mice compared with WT. These different results with 4 and 10 s analyses suggest a disruption of microarchitecture of REM in MT_1_KO; moreover, the opposing effects in NREM detectable with the 10 s analyses point out the opposing effects of MT_1_ and MT_2_ on NREM sleep: while MT_1_KO have an increase in NREM, the MT_2_KO have a decrease.

Interestingly, MT_1_KO also show an impairment at the level of dark-light cycle of the REM sleep: the quantity of REM is the same in the dark and light periods, suggesting the involvement of this receptor also in the circadian regulation of REM sleep.

Collectively, as summarized in Table 2, the study of the 24-h sleep/wake cycle in melatonin receptors knockout mice indicates that MT_1_ receptors are mostly involved in REM sleep regulation while MT_2_ receptors in NREM sleep.

**Table 2:** 24-h sleep/wake stages in MT_1_KO, MT_2_KO, and MT_1_/MT_2_KO mice.

	**NREM sleep duration**	**REM sleep duration**	**Wakefulness duration**
MT_1_KO	ø (4 s epochs) ↑ (10 s epochs)	↓	ø (4 s epochs) ↓ (10 s epochs)
MT_2_KO	↓	ø	↑
MT_1_/MT_2_KO	ø	ø	↑

*Data obtained from Comai et al. (53). ↓, decrease; ↑, increase; ø, no change; by scoring sleep/wake stages using both 4 and 10 s epochs unless otherwise specified*.

### Localization of Melatonin Receptors in Brain Regions Involved in Sleep Regulation

The SCN is the pacemaker of the circadian rhythms in the body, including the sleep-wake cycle. Both MT_1_ and MT_2_ receptors have been reported at the level of the SCN; however, while the presence of MT_1_ receptors has been demonstrated with several techniques such as RT-PCR, *in-situ* hybridization and immunohistochemistry (65–67), the data on the presence of MT_2_ receptors are not yet so clear and points only to a very low expression (65, 67, 68). Our laboratory has shown that MT_2_ receptors are located in critical areas for sleep functions. From rostral to caudal, strong, selective MT_2_ immunoreactivity of neuronal cell bodies and proximal dendrites was consistently observed in key brain regions: the septum, CA_2_ layers of the hippocampus, supraoptic nucleus, reticular nucleus of the thalamus, red nucleus, substantia nigra pars reticulata, oculomotor nuclei, and ventral tegmental nucleus (65). Moderate MT_2_ immunoreactivity was also seen in the ventral pallidum, internal globus pallidus, other sectors of the hippocampus (e.g., the dentate gyrus), paraventricular nucleus of the hypothalamus and inferior colliculus (65).

The reticular thalamus (RT) is a small area whose activation promotes NREM sleep by connecting deeper brain structures to cortex via thalamo-cortical pathways. RT generates the classic silent/burst rhythmic activity during episodes of NREM sleep (69–71). During episodes of NREM sleep, RT neurons discharge in a slow, rhythmic, burst-firing mode that is transmitted to thalamic relay nuclei and modulated by corticothalamic inputs, resulting in a widespread synchronization across neuronal assemblies (72, 73). In rats, the selective MT_2_ receptor partial agonist UCM765 induces at the level of RT neurons a rhythmic synchronized burst activity separated by periods of silence, characterized by an increased percentage of spikes in burst, an increase in mean spike per burst and a decrease in mean interburst time (34). Since this rhythmic activity promotes NREM sleep, MT_2_ receptors may thus be viewed as a key component in sleep regulation. Of note, the activation of RT neurons by UCM765 is MT_2_ receptor-mediated, since the local infusion of 4P-PDOT blocked the effects of the drug upon the neurons, and is sufficient to promote NREM sleep. Indeed, when UCM765 is injected in a brain region not primarily involved in sleep regulation but containing MT_2_ receptors such as the substantia nigra pars reticulate, no effects on NREM sleep has been observed (34).

Recently, Sharma et al. (74) found in mice that orexin neurons in the perifornical lateral hypothalamus (PFH) express MT_1_ but not MT_2_ receptors. Orexins, also known as hypocretins, are neuropeptides synthesized in the brain exclusively by neurons in the lateral hypothalamic area that makes excitatory connections to all of the arousal-promoting nuclei. Orexins are thus a crucial neurotransmitter in promoting wakefulness, and indeed melatonin injected at the level of PFH was able to induce sleep (74). Following this finding, Sharma et al. (74) claimed that melatonin via MT_1_ receptors in the PFH may induce sleep. It is our opinion that this claim requires further proof-of-concept studies (75), but MT_1_ receptors present in the PFH are likely to contribute to effects of melatonin upon the sleep-wake cycle.

We also found MT_1_ receptors at the level of 5-HT neurons in the dorsal raphe (65), and the lack of MT_1_ receptors in MT_1_KO mice impaired the physiological light-dark fluctuation of a subpopulation of dorsal raphe 5-HT neurons (76). Monoaminergic neurons fire at a steady rate during wakefulness, decrease their firing during NREM sleep, and are virtually silent during REM sleep (2). Future studies are thus warranted to examine whether MT_1_ receptors present on 5-HT neurons are involved in the modulation of sleep.

### MT_1_ and MT_2_ Receptors and Sleep Circuits: Possible Interactions

It is important at this point to improve our understanding of how the MT_1_ and MT_2_ receptors play their roles in the complex sleep circuitry composed of different brain nuclei and receptors.

The neural circuits that generate arousal and sleep (both NREM and REM) remain to be completely elucidated.

Humans are diurnal mammals, with a circadian clock that promotes wakefulness during the day. Sleep timing is phase-linked to intrinsic circadian rhythm-controlled temperature rhythms as well as extrinsic light and dark signaling (77). Homeostasis is another sleep regulator, meaning that the decrease of sleep for one night induces an increase in deep sleep quantity and quality the following night.

The manner in which the brain alternates cycles of NREM and REM remains unknown; however, a prominent role for melatonin receptors can be hypothesized. The melatonin receptors MT_1_ and MT_2_ are both present at the level of retina, but MT_2_ mRNA seems to be absent in retinal ganglion cells (78). The retinohypothalamic tract, which contains the intrinsically photosensitive retinal ganglion cells (ipRGC) and the photopigment melanopsin, inputs directly and monosynaptically to the SCN, an area rich in MT_1_ and MT_2_. Circadian signals from the SCN are transmitted sequentially to the paraventricular nuclei (PVN), intermediolateral nucleus of the spinal cord (IML), superior cervical ganglion (SCG), and finally the pineal gland (79). Bilateral SCN lesion abolishes circadian rhythms of melatonin synthesis and secretion, demonstrating that the SCN is the melatonin rhythm generator (80). The pineal gland produces melatonin when stimulated by the SCN glutamatergic neurons (in response to the darkness) (79). MLT is then released into the bloodstream through which it reaches every organ in the body, including the brain where it interacts with MT_1_ and MT_2_ located in the NREM areas (including RT) or REM area [including locus coeruleus (LC) and lateral hypothalamus (LH)]. These areas regulate in concert the different sleep cycling. It may be hypothesized that the peak of melatonin between 12 and 3 a.m. may desensitize or down-regulate its own receptors, generating a differential expression and/or sensitivity of MT_1_ (REM sleep) and MT_2_ (NREM sleep) that may in their turn generate a kind of rhythmic balance between NREM, REM and wakefulness. In support of this theory, it has been shown that MT_2_ receptors desensitize quickly after melatonin exposure (81).

Melatonin stimulates the brain's MT_2_ receptors in the NREM sleep-activating regions of the brain: the reticular thalamus and the preoptic areas, including both the ventrolateral preoptic area (vlPO) and the median preoptic area (MNPO) (34, 65). Specifically, the MNPO appears to regulate the firing activity of the vlPO (82). During the transition from wakefulness to sleep, the MNPO—which specifically contains neurons that fire during SWS and paradoxical or REM sleep, with slow discharging activity <5 Hz—begin to fire not before, but after, sleep onset, with a gradual increase in discharge rate (83).

During NREM sleep, two nuclei are particularly active: the RT, containing melatonin MT_2_ and GABA receptors and responsible for thalamocortical input to the prefrontal cortex (showing synchronized activity during NREM); and the ventrolateral preoptic area (vlPAG), containing GABA and galanin receptors, and inhibiting noradrenergic, serotonergic, cholinergic, histaminergic, and hypocretinergic neurons. These nuclei play a role in the “reciprocal inhibitory” model of the sleep–wake switch. In particular, during NREM sleep, the vlPO sends inputs that reduce the activity of the orexinergic arousal system and the monoamine nuclei [including the Ventral tegmental area (VTA) containing dopamine (DA) neurons, the dorsal raphe (DR) containing serotonin (5-HT) neurons, and the LC containing norepinephrine (NE) neurons] by releasing the inhibitory neurotransmitters GABA and galanin. As a feedback mechanism, vlPO neurons receive reciprocal inputs from the arousal nuclei including the VTA, DR, and LC; the vlPO also receives input from the histaminergic tuberomammillary nucleus (TMN) (84).

People suffering from fatal familial insomnia (FFI) show thalamic disruption that inactivates their ability to sleep, which is paralleled by a dysfunction in melatonin production (85). As mentioned before, the RT neurons discharge in burst activity exclusively during NREM, and thalamocortical pathways project this synchronous burst activity, intermingled with periods of silence, onto the cortex. This rhythmic firing activity generates the synchronized EEG pattern typical of SWS, which produces disconnection between the cortex and the outside world (86). Remarkably, the RT is also rich in melatonin MT_2_ receptors, which are likely activated at the beginning of NREM sleep (34). These receptors, which are contribute to the generation of the characteristic bursts that, through the thalamo-cortical pathways, produce the classical silent/burst activity in the PFC. Conversely, during wakefulness, the RT and thalamocortical neurons are depolarized by inputs from the reticular activating system of the brainstem, and discharge instead with a tonic activity [adapted from Purves et al. (87)].

On the other hand, REM sleep is regulated by other brain areas. The vlPAG is a putative “REM ON” nucleus, switching the brain to the REM sleep mode. During REM, the sublateral nucleus (SLD), the basal forebrain (BF), and the lateral tegmentum/ pedunculopontine tegmentum (LDT/PPT, rich in acetylcholine receptors) and the ventromedial medulla (VM) neurons become particularly active.

Many researchers have hypothesized that REM sleep is mediated mostly through cholinergic neurons located in the LDT/PPT. These neurons are active during REM sleep and generate the cortical activation and atonia typical of this sleep stage, and are inactive during NREM sleep. Indeed, LDT/PPT neurons send inputs to the ventromedial medulla (VM), which inhibits motor neurons by releasing GABA and glycine into the spinal and brainstem motor neurons, producing atonia. LDT/PPT neurons are also the main source of acetylcholine (Ach) to the thalamus: activation of this ACh pathway depolarizes thalamic neurons, generating the cortical activation associated with REM sleep and dreaming. Other nuclei important for REM sleep regulation are: (1) the sublaterodorsal nucleus (SDL) which produces GABA and glutamate and projects to the glycinergic/GABAergic premotor neurons in the ventromedial medulla and ventral horn of the spinal cord, and through these circuits likely inhibits motor neurons during REM sleep; (2) the melanin-concentrating hormone (MCH)-containing neurons that fire during REM sleep and decrease their activity during NREM sleep and wakefulness [Saper et al. (88); reviewed in España and Scammell (62)]; and (3) LC neurons that fire as a function of vigilance and arousal displaying a firing of 4–6 Hz during quiet wakefulness and a sustained activation during alertness or stress. LC NE firing decreases markedly during NREM and is completely silent during REM sleep (89, 90).

Interestingly, we found that the daily circadian changes of LC NE neural activity are blunted in MT_1_KO mice as compared with WT controls, and the bust-firing activity of LC NE neurons, that is associated with the synaptic release of the neurotransmitter (91), is significantly reduced in MT_1_KO compared with WT mice (76).

Another cholinergic nuclei that is active during REM sleep and wakefulness is the LH which contains both MT_1_ and orexin receptors (74).

**Figure 1:**
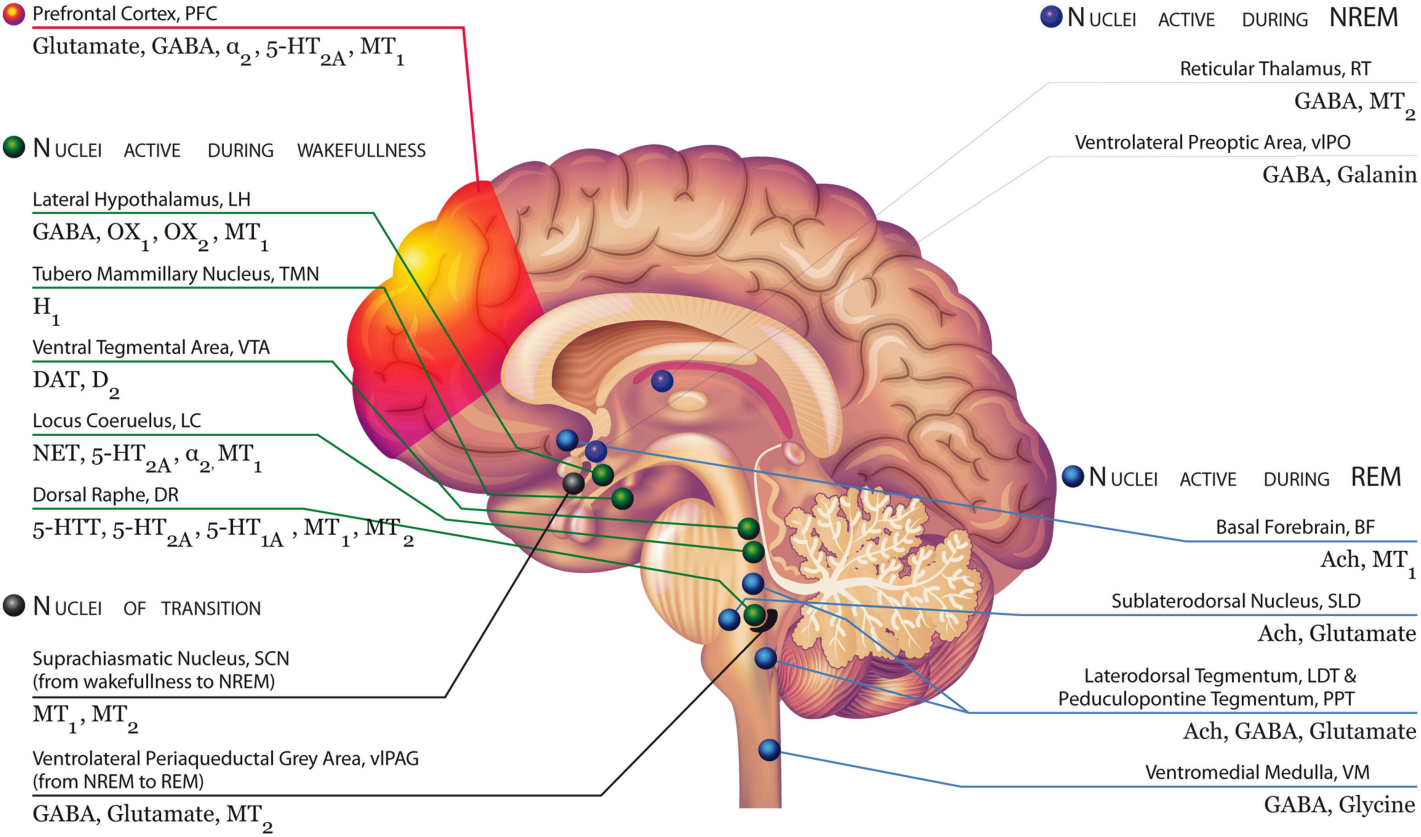
Brain areas involved in the regulation of sleep and wakefulness with their respective receptors, including MT_1_ and MT_2_ receptors Modified with permission from Atkin et al. (2). **Top left, green:** During NREM, the serotonin neurons of the Dorsal Raphe (DR), the dopaminergic neurons of the Ventral tegmental area (VTA), and the noradrenergic neurons of the Locus Coeruleus (LC) decrease their firing activity. These neurons are silent during REM. OX_1_ and OX_2_-containing orexinergic neurons of the Lateral Hypothalamus (LH) decrease their firing activity during NREM and REM. The histaminergic H_1_-containing neurons of the Tuberomammillary Nucleus (TMN) decrease their firing activity during sleep. During wakefulness, neurons of the arousal system (i.e., monoaminergic neurons, orexinergic neurons) send widespread ascending projections to the cerebral cortex, stimulating cortical desynchronization with high frequency gamma and low frequency theta rhythmic activity. **Bottom left, black:** MT_1_ and MT_2_ receptors expressed in suprachiasmatic neurons, which receive inputs directly from the retinohypothalamic tract (RHT), influenced by light and external stimuli may be likely involved in the switch from wakefulness to NREM sleep. The transition from NREM and REM is controlled by the ventrolateral periaqueductal gray area (vlPAG), containing GABA, glutamate receptors, but also melatonin MT_2_ receptors. **Top right, red:** During NREM sleep, two nuclei are particularly active: the reticular thalamus (RT), containing melatonin MT_2_ and GABA receptors, which is responsible for thalamocortical input to the prefrontal cortex (showing synchronized activity during NREM); and the ventrolateral preoptic area (vlPAG), containing GABA and galanin receptors. They inhibit noradrenergic, serotonergic, cholinergic, histaminergic, and hypocretinergic neurons. These nuclei play a role in the “reciprocal inhibitory” model of the sleep–wake switch. **Bottom right, blue:** The vlPAG is a putative “REM ON” nucleus, switching the brain to the REM sleep mode. During REM, the sublateral nucleus (SLD), the basal forebrain (BF), and the lateral tegmentum/ pedunculopontine tegmentum (LDT/PPT, rich in acetylcholine receptors) and the ventromedial medulla (VM) neurons are particularly active. The BF is active in REM and wakefulness and inhibited during NREM.

However, more research, especially with selective compounds or optogenetic techniques, is required to better differentiate the role of these two receptors in sleep regulation. Figure 1 illustrates the main areas of the brain implicated in the regulation of sleep and wakefulness with their respective receptors, including MT_1_ and MT_2_.

### Melatonin Pick, Circadian Rhythms and MT_1_/MT_2_ Receptors

The plethora of studies here reported demonstrating the weak hypnotic properties of exogenous melatonin and the fact that melatonin picks in both nocturnal and diurnal animals at the same time—between 1 and 3 a.m.—(92, 93) leads us to hypothesize that melatonin is not *per se* a neuromodulator acting on sleep, but rather a pace-maker influencing circadian rhythms among which the circadian regulation of sleep in both diurnal and nocturnal animals. Melatonin likely acts as an “orchestra conductor”: when melatonin peaks (1–3 a.m.) it regulates the expression of MT_1_, MT_2_, and other non-melatonin receptors, which are those directly regulating sleep stages. On one hand, the nocturnal overexpression of MT_2_ receptors in diurnal mammalian increases the propensity to sleep by activating the neurons that trigger NREM sleep (i.e., neurons in the RT). On the other hand, in nocturnal animals, the melatonin peak would down-regulate MT_2_ receptors while up-regulating MT_1_ and other receptors involved in wakefulness, for example monoamines (76) and orexin (74) receptors.

In support of this hypothesis, Pinato et al. (94) found that in the diurnal primate *Sapajus apella*, MT_1_ and MT_2_ receptors displayed different reciprocal patterns of expression according to the light/dark cycle in four hypothalamic nuclei, with an apparent inverse expression in the SCN compared with the other three hypothalamic areas. Pinealectomized rats (54) or humans with pineal parenchymal tumors (95) that display significantly altered rhythms in circulating levels of melatonin do not necessarily show sleep impairments, but in contrast, the activation of MT_2_ receptors or the genetic deletion of either MT_1_ or MT_2_ receptors induces significant changes in sleep stages. In line, the non-selective MT_1_-MT_2_ agonist tasimelteon, which has been approved for the treatment of non-24-h sleep–wake rhythm disorder in blind people display pharmacological efficacy as a consequence of the resynchronization to a 24-h sleep-wake rhythm (96). Interestingly, this kind of hormonal circadian regulation of the receptors has also been observed for the cortisol peak (occurring early in the morning) and the response of its glucocorticoid and mineralocorticoid receptors (97).

Importantly, similar to cortisol, circulating melatonin may not only play a role in regulating the activity and expression may of its two receptors, but also the expression (98) of clock genes, which in turn regulate a plethora of different cellular functions.

The data reported in this review indicate that the MT_2_ receptor is mostly involved in sleep, and less in the regulation of circadian rhythms. In contrast, several studies suggest that the MT_1_ receptor is mostly involved in the circadian regulation of behavior.

Indeed, *in-vitro* experiments using SCN slides showed that MT_1_ receptors control the neuronal firing rate and MT_2_ receptors the phase shift-circadian rhythm of the neuronal firing (52); however, in *in-vivo* studies, a MLT injection phase shifted the SCN activity onset of WT but not of MT_1_KO mice and also accelerated the entertainment to a new light-dark cycle of WT but not of MT_1_KO mice (52, 99), suggesting that MT_1_ receptor is involved in circadian regulation.

In keeping, MT_1_KO mice show no light/dark differences in circulating corticosterone levels (76), and unlike WT and MT_2_KO mice, no light/dark differences in the duration of REM sleep (53). Finally, the abundance of MT_1_ compared with MT_2_ receptors in the SCN (65) may also suggest a prime implication of MT_1_ receptor in circadian regulation.

Further research is necessary to validate this hypothesis linking melatonin, melatonin receptors, circadian rhythms and sleep. Within this context, it will be important to investigate the pathophysiological role of the recently characterized MT1/MT2 heteromers (17), but also of possible heterooligomers between melatonin receptors and 5-HT_2c_ receptors. Notably, 5-HT_2c_ receptors are present in considerable amounts at the level of the SCN (100) and their activation also modulate clock gene expression (101).

## Conclusions and Open Questions

Melatonin is an important modulator of the sleep/wake cycle by activating MT_1_ and MT_2_ receptors, even if some authors have also hypothesized that melatonin can have MT_1_/MT_2_ receptor-independent hypnotic effects (102). Using different experimental approaches, melatonin receptors have been shown to be present in many brain areas/nuclei implicated in the control of the sleep/wake cycle. Importantly, the most recent studies indicate that the two receptor subtypes are differently expressed in regions involved in REM or NREM sleep. For example, the MT_2_ is uniquely located in the reticular thalamus, an area involved in NREM triggering. In contrast, the MT_1_ receptor is found in the PFH, involved in REM, as well as in the dorsal raphe nucleus and the locus coeruleus, which are either active, slightly active, or silent according to the wakefulness, NREM, and REM sleep stages, respectively. The neural circuits implicated in the regulation of the sleep/wake cycle have yet to be completely elucidated, and may represent an interesting target for the application of the novel technologies of optogenetics and genetic manipulation which would allow for the activation or inactivation of single receptors in specific areas. The current knowledge we have summarized here suggests that the two melatonin receptors subtypes can have either complementary or opposing effects in NREM and REM sleep, likely because of their different expression in brain areas differently implicated in the regulation of the sleep/wake cycle. These findings result mainly result from preclinical studies genetically and/or pharmacologically targeting MT_1_ or MT_2_ receptors, and partially explain the limited efficacy as hypnotics of melatonin or non-selective MT_1_/MT_2_ receptor agonists in clinical studies. While the possible role of MT_2_ receptor in modulating sleep stages has been confirmed by studies in MT_2_ receptor knockout mice and with compounds activating selectively the MT_2_ receptor subtype, research on MT_1_ receptors is still limited to findings in MT_1_ receptor knockout mice. The development of selective ligands for the MT_1_ receptor subtype will allow us to test their effects upon the sleep/wake cycle, thus increasing our understanding of the neurobiological role of both MT_1_ and MT_2_ receptors in sleep.

Most preclinical research investigating the potential hypnotic effects of selective MT_2_ agonists/partial agonists has been conducted following only one or a few injections of the drug. No studies have evaluated the effects of a chronic treatment with these different melatonergic compounds on the sleep/wake cycle. This is particularly noteworthy since hypnotics are often prescribed in humans for long periods.

Another important issue arising from the reviewed literature is the importance of considering the time of administration of melatonergic compounds. Comparing preclinical and clinical studies, in humans the treatment has been done early or late (before going to sleep) during the day, and in animals during the light (inactive) or dark (active) phase of the day. Given the circadian variations in the endogenous levels of melatonin and likely in the expression of the two melatonin receptors, it is not surprising that different and/or apparently contrasting findings have been described. Therefore, chronopharmacology should become a *leitmotif* when discussing the potential implications of the novel findings linking the melatonin system to sleep but also to wider biological/pharmacological issues.

The history of pharmacology indeed has taught us that receptor-selective ligands are superior to the respective neurotransmitter itself. For example, serotonin or the precursor tryptophan is less effective than SSRIs for depression or 5-HT2A antagonists for psychosis. Similarly, selective MT_1_ or MT_2_ ligands may be therapeutically more effective than melatonin in the treatment of sleep disorders.

In conclusions, given the lack of medications specifically registered for treating either NREM or REM sleep disorders and the fact that MT_1_ and MT_2_ receptors seem to modulate the two sleep stages differently, the future development of selective MT_1_ or MT_2_ receptor ligands may help to answer this medical need that afflicts a considerable percentage of the population in industrialized countries.

## Author Contributions

GG and SC conceived the study, collected data, and wrote the review.

### Conflict of Interest Statement

GG is an inventor and assignee of patents for selective melatonin ligands. The remaining author declares that the research was conducted in the absence of any commercial or financial relationships that could be construed as a potential conflict of interest.
